# Oestrogen receptor-*α* contributes to the regulation of the hedgehog signalling pathway in ER*α*-positive gastric cancer

**DOI:** 10.1038/sj.bjc.6605517

**Published:** 2010-01-19

**Authors:** C Kameda, M Nakamura, H Tanaka, A Yamasaki, M Kubo, M Tanaka, H Onishi, M Katano

**Affiliations:** 1 Department of Cancer Therapy and Research, Graduate School of Medical Sciences, Kyushu University, Fukuoka, Japan; 2 Department of Surgery and Oncology, Graduate School of Medical Sciences, Kyushu University, Fukuoka, Japan

**Keywords:** gastric cancer, hedgehog signalling pathway, Sonic hedgehog, oestrogen receptor-*α*, proliferation

## Abstract

**Background:**

Oestrogen receptor-alpha (ER*α*) is highly expressed in diffuse-type gastric cancer and oestrogen increases the proliferation of ER*α*-positive gastric cancer. However, a detailed mechanism by which oestrogen increases the proliferation of these cells is still unclear.

**Methods:**

We used 17-*β*-oestradiol (E2) as a stimulator against the ER*α* pathway. Pure anti-oestrogen drug ICI 182 780 (ICI) and small interfering RNA against ER*α* (*ERα* siRNA) were used as inhibitors. Cyclopamine (Cyc) was used as the hedgehog (Hh) pathway inhibitor. Two human ER*α*-positive gastric cancer cells were used as target cells. Effects of the stimulator and inhibitor on E2-induced cell proliferation were also examined.

**Results:**

In ER*α*-positive cells, E2 increased not only cell proliferation but also one of the ligands of the Hh pathway, Shh expression. 17-*β*-Oestradiol-induced cell proliferation was suppressed by ICI, *ERα* siRNA or Cyc. The increased expression of Shh induced by E2 was suppressed by ICI and *ERα* siRNA but not by Cyc. Furthermore, recombinant Shh activated the Hh pathway and increased cell proliferation, whereas anti-Shh antibody suppressed E2-induced cell proliferation. When a relationship between ER*α* and Shh expressions was analysed using surgically resected gastric cancer specimens, a positive correlation was found, suggesting a linkage between the ER*α* and Hh pathways.

**Conclusion:**

Our data indicate that activation of the ER*α* pathway promotes cell proliferation by activating the Hh pathway in a ligand-dependent manner through Shh induction of ER*α*-positive gastric cancer.

Gastric cancer is one of the most lethal of all malignancies (Parkin, 2001). In particular, patients with unresectable, metastatic or recurrent gastric cancer have only few therapeutic options. Thus, it is urgent to develop novel therapeutics for gastric cancer.

Gastric cancers are classified into two major histological types using the criteria of Lauren, namely intestinal type and diffuse type (Lauren, 1965). The carcinogenic pathway of intestinal-type gastric cancer develops through several sequential stages with *Helicobacter pylori* infection, followed by chronic gastritis, atrophic gastritis and intestinal metaplasia (Yuasa, 2003), whereas the diffuse type is believed to be derived from hyperplastic stem or precursor cells (Ming, 1998). It is considered that diffuse-type gastric cancer has a higher malignant potential such as invasion and metastasis, compared with intestinal-type gastric cancer.

Oestrogen has various physiological functions, such as normal cell growth and differentiation in many target tissues. Oestrogen is produced not only from the ovary but also from extra-ovarian tissues, that is, from the skin, brain, testis, adipose tissues and vascular smooth muscle (Ackerman *et al*, 1981; Leshin *et al*, 1981; Brodie and Inkster, 1993; Lephart, 1996; Ueyama *et al*, 2002). The biological actions of oestrogen are mediated through two specific receptors (oestrogen receptor, ERs), ER*α* and ER*β*, which belong to the nuclear receptor superfamily. Oestrogen-bound ERs bind as homodimers or heterodimers to a specific DNA sequence known as the oestrogen-responsive element (ERE), and regulate the transcription of target genes (Matthews and Gustafsson, 2003). As oestrogen is a stimulant for the initiation and promotion of breast cancer, and ER*α* can be expressed in gastric cancer cells, it has been suggested that the ER*α* pathway may have a role in the progression of gastric cancer (Harrison *et al*, 1989b; Wu *et al*, 1994; Karat *et al*, 1999; Takano *et al*, 2002; Chandanos *et al*, 2008). In gastric cancer, ER*α* expression is higher in the diffuse type than in the intestinal type (Kitaoka, 1983; Tokunaga *et al*, 1986; Matsui *et al*, 1992; Zhao *et al*, 2003), and oestradiol (E2) enhances gastric cancer cell proliferation *in vitro* (Matsui *et al*, 1992; Takano *et al*, 2002). On the basis of these observations, several clinical trials using a partial oestrogen antagonist, tamoxifen (TAM), have been conducted for the management of ER*α*-positive gastric cancer patients. However, the results have not been consistent and the utility of ER*α* for the treatment of gastric cancer is still controversial (Harrison *et al*, 1989a, 1989b; Koullias *et al*, 2003).

The hedgehog (Hh) signalling pathway has a crucial role in embryonic development, tissue regeneration and carcinogenesis. Of the three Hh ligands, namely Sonic Hh (*Shh*), Indian Hh (*Ihh*) and Desert Hh (*Dhh*) (Pasca di Magliano and Hebrok, 2003; Lum and Beachy, 2004; Briscoe and Therond, 2005; Hooper and Scott, 2005; Katoh and Katoh, 2005), Shh is exclusively expressed in the acid-producing parietal cells in both human and murine stomach, and is believed to be a regulator of gastric fundic gland differentiation and mutation (Ramalho-Santos *et al*, 2000; van den Brink *et al*, 2001, 2002; Dimmler *et al*, 2003). Pharmacological inhibition of Shh signalling in the adult stomach causes loss of parietal cells (gastric atrophy) and subsequent disruption of glandular differentiation (van den Brink *et al*, 2002). Furthermore, Shh is strongly implicated in maintaining stem cell niches in the adult stomach (Katoh and Katoh, 2006). Recently, ligand-dependent activation of the Hh pathway, especially due to an aberrant expression of Shh, has been detected in various cancers including gastric cancer (Berman *et al*, 2003; Watkins *et al*, 2003; Karhadkar *et al*, 2004; Sanchez *et al*, 2004; Sheng *et al*, 2004). Overexpression of Shh leads to carcinogenesis through the aberrant activation of the Hh pathway (Berman *et al*, 2003; Thayer *et al*, 2003; Cengel, 2004; Sanchez *et al*, 2004). It is also believed that Shh has an important role during the progression of gastric cancer, because Shh expression is much higher in the diffuse type than in the intestinal type in human gastric cancer (Ma *et al*, 2005, 2006).

It is noteworthy that our previous study demonstrated a positive correlation between Hh pathway activation and ER*α* status (Kubo *et al*, 2004), and suggested that ER*α* could regulate Hh pathway activation in ER*α*-positive breast cancer (Koga *et al*, 2008). It has also been shown that the Hh pathway can be a useful therapeutic target against breast cancer and gastric cancer (Kubo *et al*, 2004; Yanai *et al*, 2007). From these results, we hypothesised that stimulation of the ER*α* pathway induces Shh expression, activates the Hh pathway and consequently promotes cell proliferation in ER*α*-positive gastric cancer cells. The ER*α* pathway could be a possible therapeutic target for patients with ER*α*-positive gastric cancer.

## Materials and methods

### Cell culture, reagents and antibodies

We prepared eight gastric cancer cell lines, KATOIII, NCI-N87, MK-1, AGS, MKN45, Hs746T, SNU-16 and MKN74. These were maintained at 37°C under a humidified atmosphere of 5% CO_2_ and 95% air in RPMI1640 medium (Life Technologies, Grand Island, NY, USA) supplemented with 10% fetal bovine serum (FBS; Life Technologies) and antibiotics (100 Units ml^−1^ penicillin and 100 *μ*g ml^−1^ streptomycin; Meijiseika, Tokyo, Japan). Cells were incubated for 24 h in phenol-red-free minimum essential medium (MEM; Invitrogen, Carlsbad, CA, USA) without FBS before all experiments (termed oestrogen starvation). Thereafter, cells were principally cultured in MEM supplemented with 5% dextran-coated charcoal-treated FBS (DCC-FBS-MEM) in a humidified 95% air and 5% CO_2_ atmosphere. 17-*β*-Oestradiol was purchased from Sigma (Deisenhofen, Germany). The ER pathway antagonist, ICI 182 780 (ICI), was purchased from Tocris Cookson Ltd. (Ellisville, MO, USA). Cyclopamine (Cyc) was purchased from Toronto Research Chemicals (North York, ON, Canada). Both ICI and Cyc were diluted in 99.5% ethanol as a stock solution, and stored at −30°C. Recombinant human Shh N-terminal peptide (rhShh) was purchased from R&D Systems (Minneapolis, MN, USA). Rat anti-Shh NH_2_-terminal peptide antibody (*α*Shh-Ab) was purchased from R&D Systems.

### Expression vectors

cDNAs encoding human ER*α* were cloned into the pSG5 expression vector as described previously (Green *et al*, 1988; Kato *et al*, 2002). This cDNA and ERE-tk-luc, a single consensus ERE upstream of luciferase, were kindly provided by Dr Norio Wake (Kyushu University, Fukuoka, Japan). pRL-SV40 was purchased from Promega (Madison, WI, USA).

### Reverse transcription–PCR

Total RNA was extracted from cultured cells by the guanidinium thiocyanate–phenol–chloroform single-step method. For the reverse transcription (RT) reaction, pd(N)_6_ Random Hexamer (GE Healthcare UK Ltd, Buckinghamshire, England) was used for priming. The *ERα* forward (5′-CAG GGG TGA GTG GGG TC-3′) and reverse (5′-ATG CGG AAC CGA GAT GAT-3′) primers yielded a 483-bp product. *Gli1* forward (5′-TCT GCC CCC ATT GCC CAC TTG-3′) and reverse (5′-TAC ATA GCC CCC AGC TAC CTC-3′) primers yielded a 480-bp product. The *Shh* forward (5′-CGC ACG GGG ACA GCT CGG AAG T-3′) and reverse (5′-CTG CGC GGC CCT CGT AGT GC-3′) primers yielded a 477-bp product. The *Ptch1* forward (5′-CGG CGT TCT CAA TGG GCT GGT TTT-3′) and reverse (5′-GTG GGG CTG CTG TCT CGG GTT CG-3′) primers yielded a 376-bp product. Glyceraldehyde-3-phosphate dehydrogenase (*GAPDH*) forward (5′-CCA CCC ATG GCA AAT TCC ATG GCA-3′) and reverse (5′-TCT AGA CGG CAG GTC AGG TCC ACC-3′) primers yielded a 593-bp product. The amplification conditions comprised an initial denaturation for 2 min at 95°C, followed by 35 cycles of 94°C for 30 s, 58°C for 30 s and 72°C for 1 min. The amplification of each gene was in the linear range. The RT–PCR products were separated on ethidium bromide-stained 2% agarose gels. Semi-quantitative analysis was carried out with a Molecular Imager FX Pro (Bio-Rad Laboratories, Hercules, CA, USA).

### Real-time RT–PCR

Total RNA was extracted using the RNeasy mini kit (Qiagen, Valencia, CA, USA) and quantified by spectrophotometry (Ultrospec 2100 Pro; Amersham Pharmacia Biotech, Cambridge, UK). RNA (700 ng) was treated with DNase, and reverse transcribed to cDNA using the Quantitect Reverse Transcription Kit (Qiagen) according to the manufacturer's protocol. Reactions were run with SYBR Premix Ex Taq (Takara Bio Inc., Otsu, Japan) on a DNA Engine Opticon 2 System (MJ Research, Waltham, MA, USA). pShh-GFP or pGli1 was serially diluted in 10-fold increments and amplified with the primer pairs to generate a standard curve for *Shh* or *Gli1*. Standard curves for *β-actin* were generated using cDNA from MK-1 cells treated with E2 for 8 h. Each sample was run in triplicate. All primer sets amplified fragments <200 bp long. The sequences of the primers used were as follows: *β-actin*, forward, 5′-TTG CCG ACA GGA TGC AGA AGG A-3′, reverse, 5′-AGG TGG ACA GCG AGG CCA GGA T-3′ *ERα*, forward, 5′-GGA GGG CAG GGG TGA A-3′, reverse, 5′-GGC CAG GCT GTT CTT CTT AG-3′ *pS2*, forward, 5′-CAT GGA CGT CCC TCC AGA AGA G-3′, reverse, 5′-CTC TGG GAC TAA TCA CCG TGC TG-3′ *Shh*, forward, 5′-GTG TAC TAC GAG TCC AAG GCA C-3′, reverse, 5′-AGG AAG TCG CTG TAG AGC AGC-3′ and *Gli1*, forward, 5′-GGT TCA AGA GCC TGG GCT GTG T-3′, reverse, 5′-GGC AGC ATT CTC AGT GAT GCT G-3′. The amount of each target gene in a given sample was normalised to the level of *β-actin* in that sample.

### Immunoblotting

Whole-cell extraction was performed with M-PER Reagents (Pierce Biotechnology, Rockford, IL, USA) according to the manufacturer's instructions. Protein concentration was determined using the Bio-Rad Protein Assay (Bio-Rad Laboratories), and whole-cell extract (80 *μ*g) was separated by electrophoresis on a SDS-polyacrylamide gel, and transferred to Protran nitrocellulose membranes (Whatman, Dassel, Germany). Blots were then incubated overnight with GAPDH (1 : 1000), ER*α* (1 : 200) or Shh (1 : 200) primary antibody at 4°C. Blots were then incubated in HRP-linked secondary antibody (Amersham Biosciences, Piscataway, NJ, USA) at room temperature for 1 h. Immunocomplexes were detected using ECL together with the western blotting detection system (Amersham Biosciences) and visualised using a Molecular Imager FX (Bio-Rad Laboratories). Glyceraldehyde-3-phosphate dehydrogenase was used as a protein loading control.

### Dual luciferase assay

KATOIII and NCI-N87 cells in 24-well plates were transfected with plasmids with TransFast transfection reagent according to the manufacturer's instructions. Cells on each well were co-transfected with 10 ng of pRL-SV40 (Promega) and 1 *μ*g ERE-tk-Luc. After oestrogen starvation, E2 and ICI or Cyc were added to each well for 8 h, and luciferase assays were performed using the dual luciferase assay kit (Promega) according to the manufacturer's instructions. The luciferase activities were normalised to *Renilla* luciferase activity.

### Small interfering RNA against ER*α*

KATOIII and NCI-N87 cells (1.0 × 10^6^ cells) were transfected with small interfering RNA (siRNA) (100 nM) against ER*α* by lipofectamine as per the manufacturer’s instructions, and then plated in a 25-cm^2^ flask for 24 h in 10% FBS-RPMI. After oestrogen starvation, the cells were treated with E2 for 16 h, and then used for real-time RT–PCR. The following siRNAs were used: Validated Stealth RNAi against ER*α* and the Stealth RNAi-negative control (Invitrogen).

### Proliferation assay

KATOIII (5 × 10^3^ per well), NCI-N87 (1 × 10^4^ per well) and MK-1 (5 × 10^3^ per well) cells were seeded in 48-well plates in complete culture medium and were incubated overnight. After oestrogen starvation, the medium was changed to 5% DCC-FBS-MEM containing various concentrations of reagents. After 72 h of incubation, cells were harvested by trypsinisation, and viable cells were counted using a Coulter counter (Beckman Coulter, Fullerton, CA, USA).

### Clinical samples

Surgical specimens were obtained from 20 patients with diffuse-type gastric cancer and from 20 patients with intestinal type. All of the patients underwent resection at the Department of Surgery and Oncology, Kyushu University (Fukuoka, Japan), between 1996 and 2004. All 40 patients gave informed consent before surgical treatment and were enrolled into this study. All surgical specimens were frozen at −80°C, examined histopathologically and classified using the tumour-node-metastasis classification. Total mRNA of these specimens was extracted using the RNeasy mini kit (Qiagen) as per the manufacturer’s recommendation.

### Immunohistochemistry

Single-antibody detection was carried out as described previously (Kubo *et al*, 2004), with the following protocol modifications: Endogenous peroxidase activity was blocked using 3% H_2_O_2_ in methanol for 30 min at room temperature. Antigen retrieval was achieved by boiling tissue in 0.01 mol l^−1^ sodium citrate (pH 6.0) for 5 min. All primary antibodies were incubated overnight at 4°C. The primary antibodies used were Shh (1 : 50 H-160, sc-9024,) and ER*α* (1 : 50 F-10, sc-8002) (Santa Cruz Biotechnology, Santa Cruz, CA, USA). Secondary antibodies (Shh rabbit anti-IgG; ER*α*, mouse anti-IgG; Nichirei Co., Ltd, Tokyo, Japan) were applied for 1 h at room temperature. Protein was detected by brown pigmentation using the standard 3, 3′-diaminobenzidine (DAB) protocol. Slides were lightly counterstained with haematoxylin. Negative controls were obtained in all cases by omitting the first antibodies. All primary antibodies had been previously tested for immunohistostaining.

### Statistical analysis

Student's *t-*test was used for statistical analysis, unless indicated otherwise. The correlation of clinicopathological features between the ER*α* and Shh expression was analysed by the Mann–Whitney *U-*test. All calculations were carried out using the StatView 5.0 J software (Abacus Concepts, Berkeley, CA, USA). Single asterisk denotes *P*<0.05; *P*-values <0.05 were considered significant.

## Results

### Oestrogen activates the ER*α* pathway and induces increased proliferation

**Figure 1:**
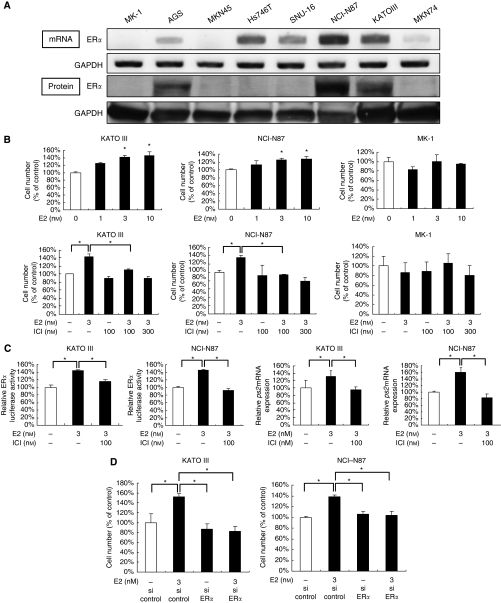
E2 activates the ER*α* pathway and induces increased proliferation in ER*α*-positive gastric cancer cells. (**A**) Both mRNA (upper panel) and protein (lower panel) expression of ER*α* were increased in KATOIII and NCI-N87, as shown by RT–PCR and western blotting. GAPDH was used as a loading control. (**B**) Cell proliferation in the presence of E2 alone (upper panel) or in combination with ICI (lower panel) in KATOIII, NCI-N87 and MK-1 cells. Cells were cultured in 5% DCC-FBS-MEM (DCC-FBS) for 72 h. (**C**) A dual luciferase assay was performed 8 h after treatment with the indicated reagents (left two panels). Relative *pS2* mRNA expression in KATOIII and NCI-N87 was examined by real-time RT–PCR (right two panels). (**D**) Cell proliferation in KATOIII and NCI-N87 cells transfected with *ERα*-siRNAs or control siRNA. After transfection with *ERα* siRNA or control siRNA, KATOIII and NCI-N87 cells were treated with or without E2 for 72 h. Results are expressed as mean±s.d. ^*^P<0.05.

We examined ER*α* expression in eight human gastric cancer cell lines. Five cell lines showed *ERα* expression at the mRNA level (Figure 1A), and three of the five cell lines, namely KATOIII, NCI-N87 and AGS cells, also showed detectable ER*α* expression at the protein level (Figure 1A). Thus, we selected KATOIII (diffuse type) and NCI-N87 (intestinal type) cells as ER*α*-positive cells, and selected MK-1 cells (diffuse type) as ER*α*-negative cells. We then examined whether the ER*α* pathway has a role in the proliferation of ER*α*-positive cells. As we expected, E2 increased the proliferation of ER*α*-positive but not negative cells in a dose-dependent manner (Figure 1B). This E2-induced cell proliferation was completely inhibited by ICI 182 780 (100 nM). ICI did not affect cell proliferation without supplementation of E2 (Figure 1B). On the basis of these preliminary data, E2 and ICI were used at a concentration of 3 and 100 nM, respectively, throughout the study. We further examined whether the ER*α* pathway is functional in these ER*α*-positive cells. Activation of the ER*α* pathway by E2 was determined with an ER*α* reporter assay and *pS2* expression, a target gene of the ER*α* pathway (Campbell-Thompson, 1997; Singh *et al*, 1997). We found that E2 increased both ER*α-*responsive reporter activity (Figure 1C) and *pS2* mRNA expression (Figure 1C) in these ER*α*-positive cells. This increased activation of the ER*α* pathway by E2 was almost completely inhibited by ICI. Finally, to confirm the contribution of the ER*α* pathway to E2-induced proliferation of ER*α*-positive cells, we silenced *ERα* mRNA expression of ER*α*-positive cells by RNA interference. Transfection of siRNA resulted in an 85% or greater knockdown of *ERα* mRNA expression (data not shown). Silencing of ER*α* did not affect cell proliferation without supplementation of E2. However, E2-induced cell proliferation was inhibited by *ERα*-siRNA (Figure 1D). These data indicate that E2 increases proliferation of ER*α*-positive gastric cancer cells through activation of the ER*α* pathway.

### Oestrogen-induced cell proliferation is suppressed by inhibition of the Hh pathway

**Figure 2:**
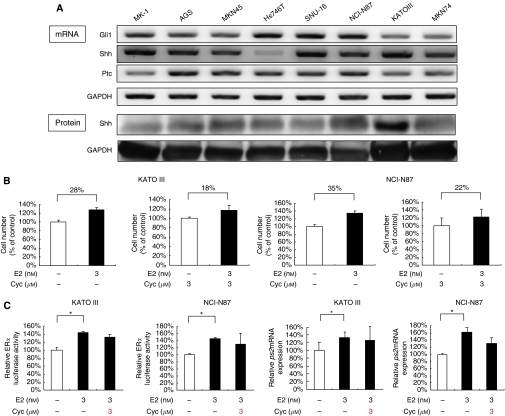
E2-induced cell proliferation is suppressed by inhibition of the Hh pathway in ER*α*-positive gastric cancer cells. (**A**) Both mRNA (upper panel) and protein (lower panel) expressions of Hh-related molecules were increased in KATOIII, NCI-N87 and MK-1 cells, as shown by RT–PCR and western blotting. GAPDH was used as a loading control. (**B**) The increasing rate of proliferation induced by E2 in the presence and absence of Hh pathway activity. (**C**) An ER*α* dual luciferase assay in KATOIII and NCI-N87 cells was performed 8 h after treatment with indicated reagents (left two panels). Relative *pS2* mRNA expression in KATOIII and NCI-N87 cells was examined by real-time RT–PCR (right two panels). Results are expressed as mean±s.d. ^*^P<0.05.

As our previous study indicated a crosstalk between the ER*α* and Hh pathways in ER*α*-positive breast cancer cells (Koga *et al*, 2008), we examined the expression of Hh-related molecules, including Shh, Ptched 1 (Ptc) and Gli1, in ER*α*-positive gastric cancer cells. Consistent with our previous report (Yanai *et al*, 2007), we found that these cells expressed Hh-related molecules at the mRNA level (Figure 2A). *Gli1* expression indicates a constitutive activation of the Hh pathway in gastric cancer cells, because *Gli1* is a target gene of the Hh pathway (Kubo *et al*, 2004). In our previous report, cyclopamine suppressed the proliferation of these cells (Yanai *et al*, 2007). To estimate a contribution of the Hh pathway to E2-induced increase in proliferation, we compared the increasing rate of proliferation induced by E2 between the presence and absence of Hh pathway activity (Figure 2B). In Kato III, the increasing rate was 28 and 18% in the presence and absence of Hh pathway activity, respectively. In NCI-N87, it was 35 and 22%, respectively. These data indicate that a block of the Hh pathway reduces the degree of E2-induced increase of proliferation. To determine the relationship between these two pathways, we examined how cyclopamine affects ER*α* pathway activation induced by E2. We found that cyclopamine did not affect E2-induced activation of the ER*α* pathway (Figure 2C). These data suggest that the Hh pathway contributes to E2-induced cell proliferation of ER*α*-positive gastric cancer cells.

### Oestrogen induces the increased Shh expression through activation of the ER*α* pathway

**Figure 3:**
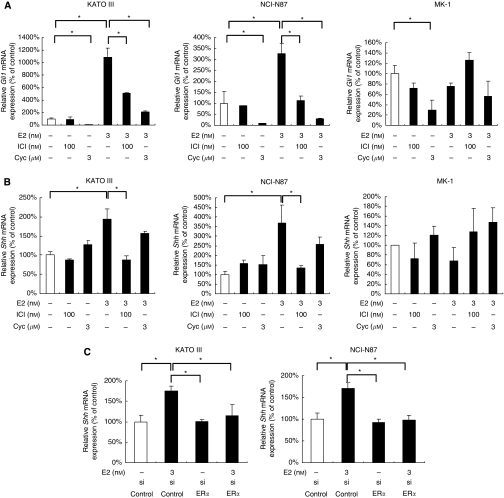
E2 upregulates Shh expression through ER*α* activation in ER*α*-positive gastric cancer cells. (**A**) *Gli1* mRNA in KATOIII, NCI-N87 and MK-1 cells treated with the indicated reagents for 8 h was examined by real-time RT-PCR. (**B**) *Shh* mRNA in KATOIII, NCI-N87 and MK-1 cells treated with the indicated reagents for 8 h was examined by real-time RT-PCR. (**C**) After transfection with *ERα* siRNA or control siRNA, KATOIII and NCI-N87 cells were treated with E2 for 8 h and then *Shh* mRNA was examined by real-time RT–PCR. Results are expressed as mean±s.d. ^*^P<0.05.

As contribution of the Hh pathway to E2-induced cell proliferation has been previously suggested, we examined whether E2 could induce Hh pathway activation in ER*α*-positive cells. Activation of the Hh pathway was determined by *Gli1* mRNA expression. We found that E2 markedly increased *Gli1* mRNA expression in ER*α*-positive cells, and that the increased *Gli1* mRNA expression was significantly suppressed by ICI. ICI alone did not affect *Gli1* mRNA expression without supplementation of E2 (Figure 3A). Cyclopamine suppressed this E2-induced *Gli1* mRNA expression more strongly than did ICI. 17-*β*-Oestradiol-induced *Gli1* mRNA expression was not found in ER*α*-negative cells. We then investigated the molecular mechanisms by which E2 activates the Hh pathway in ER*α*-positive cells. Ligand-dependent activation of the Hh pathway has been shown in gastric cancer (Berman *et al*, 2003). Therefore, we focused on an Hh pathway ligand, Shh. As expected, E2 increased *Shh* mRNA expression of ER*α*-positive cells, and this E2-induced increase was almost completely inhibited by ICI (Figure 3B). Cyclopamine did not affect E2-induced *Shh* expression. Neither ICI nor cyclopamine significantly affected *Shh* expression in ER*α*-negative cells. Finally, to confirm a contribution of the ER*α* pathway to E2-induced *Shh* mRNA expression in ER*α*-positive cells, we used ER*α*-silenced ER*α*-positive cells as target cells. Silencing of ER*α* alone did not affect *Shh* expression when there was no supplementation of E2. Importantly, E2-induced *Shh* expression was almost completely inhibited in ER*α*-silencing cells (Figure 3C). These data indicate that E2 increases Shh induction of ER*α*-positive gastric cancer cells through the activation of the ER*α* pathway.

### Oestrogen increases cell proliferation through Shh induction, followed by increased Hh pathway activation

**Figure 4:**
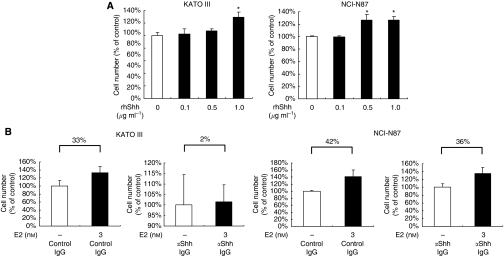
E2 increases cell proliferation through Shh induction, followed by increased Hh pathway activation. (**A**) KATOIII and NCI-N87 cells were treated with rhShh at the indicated concentrations for 72 h. rhShh was resolved in PBS containing 0.1% bovine serum albumin (BSA) as per the manufacturer's recommendation. Each control contained the same amount of BSA. Results are expressed as percentage (%) cell number to each control. (**B**) Cells were treated with anti-Shh antibody (*α*Shh-Ab, 30 *μ*g ml^−1^) or isotype-matched control IgG (Control IgG, 30 *μ*g ml^−1^) in the presence of E2 in KATOIII and NCI-N87 cells for 72 h. *α*Shh-Ab or control IgG was added to the medium at the same time as E2. The increasing rate of proliferation induced by E2 in the presence and absence of *α*Shh antibody is expressed as mean±s.d. ^*^P<0.05.

We next examined whether Shh induced by E2 could cause the proliferation of ER*α*-positive cells in a ligand-dependent manner. As mentioned above, the Hh pathway is constitutively activated in these cells. Therefore, we first investigated whether exogenous Shh could further increase the proliferation of these cells. We found that rhShh increased *Gli1* mRNA expression (data not shown) and the proliferation of these ER*α*-positive cells in a dose-dependent manner (Figure 4A). We investigated whether a neutralising antibody against Shh could suppress E2-induced proliferation of ER*α*-positive cells. Anti-Shh antibody decreased *Gli1* mRNA expression (data not shown). Anti-Shh antibody reduced the increasing rate of proliferation induced by E2 (Figure 4B). In KATO III, the increasing rate was 2 and 33% in the presence and absence of the anti-Shh antibody, respectively. In NCI-N87, it was 36 and 42%, respectively. These data indicate that the anti-Shh antibody reduces the degree of E2-induced increase of proliferation, suggesting a contribution of Shh to E2-induced proliferation. Taken together, these results suggest that Shh induced by E2 is functional and increases cell proliferation by activating the Hh pathway in a ligand-dependent manner.

### *ERα* mRNA expression positively correlates with *Shh* mRNA expression in gastric cancer tissues

**Figure 5:**
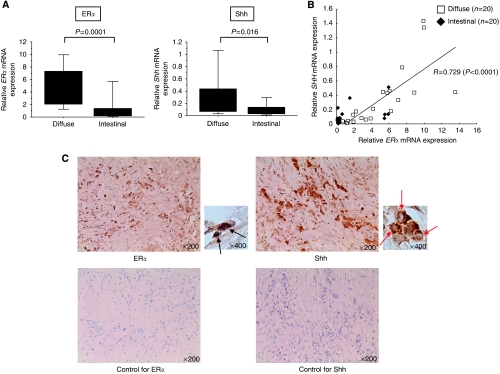
*ERα* mRNA expression positively correlates with *Shh* mRNA expression in gastric cancer tissues. (**A**) Relative *ERα* and *Shh* mRNA expressions in histological diffuse-type or intestinal-type gastric cancer are shown after normalisation to the corresponding *β-actin* mRNA expression. (**B**) Correlation with *ERα* and *Shh* mRNA expression in gastric cancer tissues. These were significantly correlated (*R*=0.729, *P*<0.0001). (**C**) The representative photographs of immunohistochemistry for ER*α* and Shh in a diffuse-type gastric cancer tissue. ER*α* expression (arrows) was observed in gastric cancer cells (upper left panels). Shh expression (arrow heads) was also observed in gastric cancer cells (upper right panels). The photographs stained with secondary antibody alone are shown as control (lower panels).

We investigated the possibility that a correlation between the ER*α* and Hh pathways exists *in vivo* using 40 surgically resected gastric cancer tissues. When 40 gastric cancer tissues were histologically divided into two groups, that is, 20 diffuse type and 20 intestinal type, no significant correlation between histological type and UICC stage was found (data not shown). The *ERα* mRNA expression was significantly higher in diffuse-type gastric cancer tissues than in intestinal-type gastric cancer tissues (*P*<0.0001; Figure 5A). Similarly, *Shh* mRNA expression was significantly higher in diffuse-type gastric cancer tissues than in intestinal-type gastric cancer tissues (*P*<0.016; Figure 5A). As expected, mRNA expression levels of *ERα* and *Shh* were tightly correlated (*P*<0.0001; Figure 5B). The correlation between *ERα* and *Shh* is significantly stronger in diffuse-type (*P*<0.0001; Supplementary Figure 1A) gastric cancer tissues than in intestinal-type gastric cancer tissues (*P*<0.0226; Supplementary Figure 1B). Expression of ER*α* and Shh was also examined immunohistochemically. Consistent with mRNA expression, both ER*α* and Shh were also highly expressed in diffuse-type gastric cancer cells (Figure 5C). In addition, histochemical analysis showed that Shh is mainly expressed by cancer cells themselves. In this study, however, we did not perform correlation analysis between these molecules at the protein level, because it was difficult to accurately estimate the expression intensity. Nevertheless, our data suggest that a novel link between the ER*α* and Hh pathways is present even in gastric cancer tissues.

## Discussion

We have shown, for the first time, a biologically significant linkage between the ER*α* and Hh pathways in ER*α*-positive gastric cancer cells. Briefly, oestrogen activates the ER*α* pathway, induces Shh production, activates Hh activation in an Shh-dependent manner and consequently increases cell proliferation in ER*α*-positive gastric cancer cells. Our data suggest that the ER*α* pathway could be a possible therapeutic target for patients with ER*α*-positive gastric cancer.

Consistent with our present data, it has already been shown that both ER*α* (Kitaoka, 1983; Tokunaga *et al*, 1986; Matsui *et al*, 1992; Zhao *et al*, 2003) and Shh (Ma *et al*, 2005; Yanai *et al*, 2007) are frequently expressed in diffuse-type gastric cancer, compared with intestinal type. Recently, it was shown that the Hh pathway might be linked to other proliferation-related signalling pathways. We have shown a crosstalk of the Hh pathway with the nuclear factor *κ*-B pathway in pancreatic cancer (Nakashima *et al*, 2006), the Wnt pathway in colonic and gastric cancer (Akiyoshi *et al*, 2008; Yanai *et al*, 2008) and the ER*α* pathway in breast cancer (Koga *et al*, 2008). Thus, we speculated that there was a linkage between the ER*α* and Hh pathways in diffuse-type gastric cancer. Our present data showed two key points in the linkage between the two pathways. One finding is that the ER*α* pathway affects the Hh pathway, because E2 induces both ER*α* pathway activation and Shh induction, followed by Hh pathway activation, and all of these phenomena were almost completely suppressed by blockade of the ER*α* pathway. The other finding is that the Hh pathway does not affect the ER*α* pathway, because neither blockade nor stimulation of the Hh pathway affected ER*α* pathway activation. A key molecule linking the ER*α* pathway with the Hh pathway is Shh, which is induced by ER*α* pathway activation. Thus, we speculate that a crosstalk between the two pathways is a one-way link from the ER*α* to the Hh pathway. The molecular mechanisms by which ER*α* pathway activation induces Shh production remain unclear.

Ligand-dependent Hh pathway activation participates in the increased proliferation of gastric cancer (Berman *et al*, 2003; Yanai *et al*, 2007). Therefore, in our study, it is not surprising that oestrogen-induced Shh induction increased the proliferation of ER*α*-positive gastric cancer cells. Our data indicate that blockade of the ER*α* pathway, such as using anti-oestrogens, may be valuable therapeutic tools for patients with ER*α*-positive gastric cancer. Interestingly, several clinical randomised controlled studies have already been performed using the selective ER modulator, TAM. Although ER status is an independent poor prognostic factor in gastric cancer (Harrison *et al*, 1989b; Matsui *et al*, 1992), the results of clinical randomised controlled studies disappointed us (Harrison *et al*, 1989a, 1989b). Nevertheless, our present data still indicate a possibility of an ER*α* pathway blocker as a therapeutic tool for patients with ER*α*-positive gastric cancer. When TAM was used at concentrations (1 *μ*M) that do not affect cell proliferation of ER*α*-negative MK-1 cells (Supplementary Figure 2B), TAM did not significantly suppress both E2-induced Hh activation and E2-induced cell proliferation of ER*α*-positive gastric cancer cells (Supplementary Figure 2A). In contrast, ICI, at a concentration (100 nM) that does not affect cell proliferation of MK-1cells, almost completely suppressed both E2-induced Hh activation and E2-induced cell proliferation. It is well known that TAM can block only one domain, that is, activating function 2 (AF-2), among two distinct ER*α* activation domains (Ali and Coombes, 2002), whereas ICI can block both of the activation domains. In addition, it has been reported that TAM seems to function as a partial agonist in uterine tissues (Kedar *et al*, 1994). These findings indicate a possibility that different anti-oestrogens may display different tissue-specific actions. Our data indicate that the development of clinically available antagonists that can sufficiently block oestrogen-induced cell proliferation is promising for management of patients with aggressive diffuse-type gastric cancer. Our data also indicate that a combination of Hh inhibitors and anti-oestrogens may be more effective against ER*α*-positive gastric cancer. Furthermore, molecules contributing to E2-induced Shh induction may be novel therapeutic candidate molecules for the management of gastric cancer patients.

## Conflict of interest

The authors declare no conflict of interest.
