# Supplementary material for: Oestrogen receptor-α contributes to the regulation of the hedgehog signalling pathway in ERα-positive gastric cancer
Source: Br J Cancer. 2010 Jan 19;102(4):738–47. doi: 10.1038/sj.bjc.6605517 (PMC2837575; doi:10.1038/sj.bjc.6605517)
Supplement: Supplementary Figures [file 6605517x1.ppt]

## Slide 1
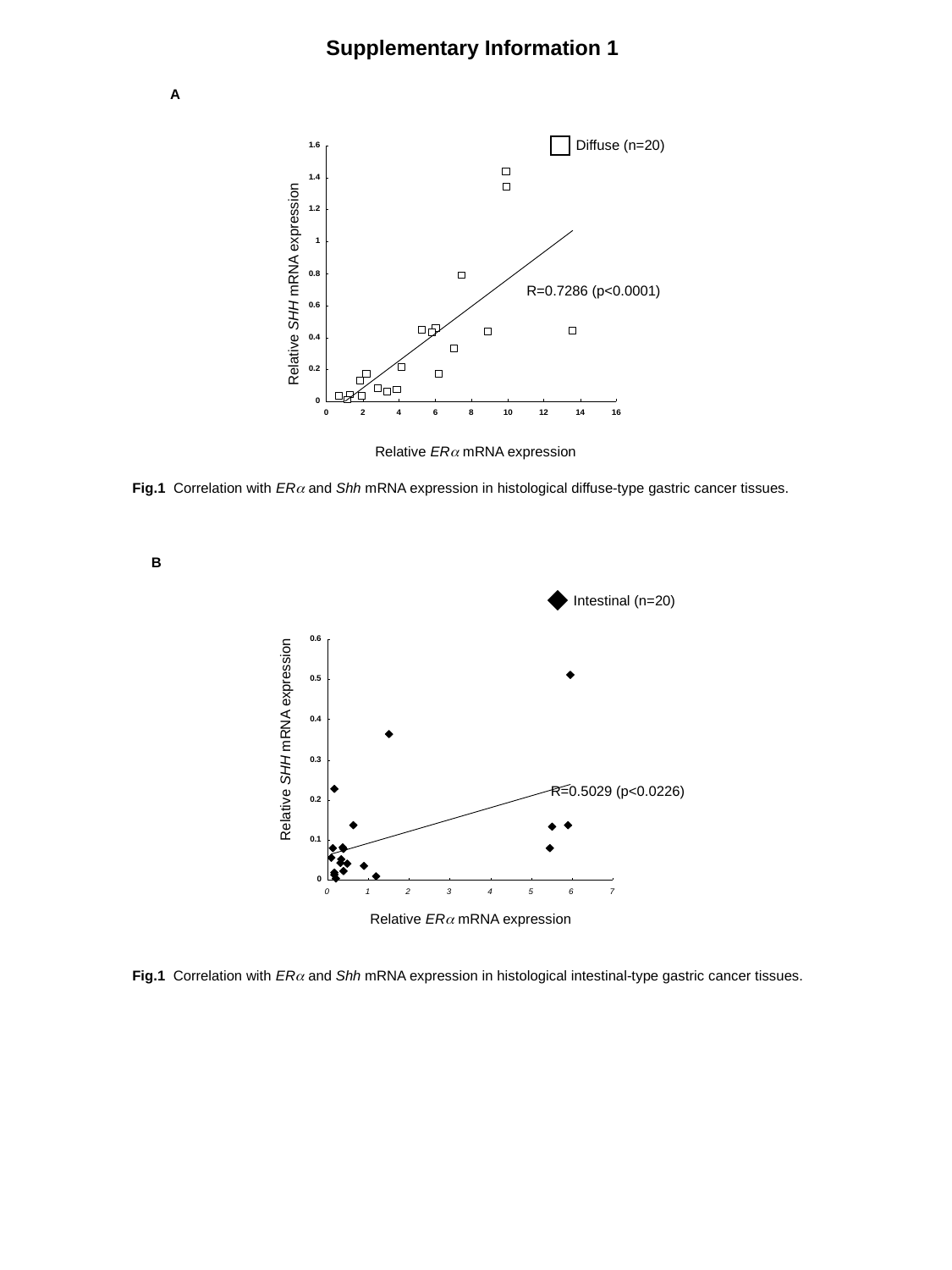

Supplementary Information 1
A
Diffuse (n=20)
R=0.7286 (p<0.0001)
Relative SHH mRNA expression
Relative ERα mRNA expression
Fig.1 Correlation with ERα and Shh mRNA expression in histological diffuse-type gastric cancer tissues.
B
Intestinal (n=20)
Relative SHH mRNA expression
R=0.5029 (p<0.0226)
Relative ERα mRNA expression
Fig.1 Correlation with ERα and Shh mRNA expression in histological intestinal-type gastric cancer tissues.

## Slide 2
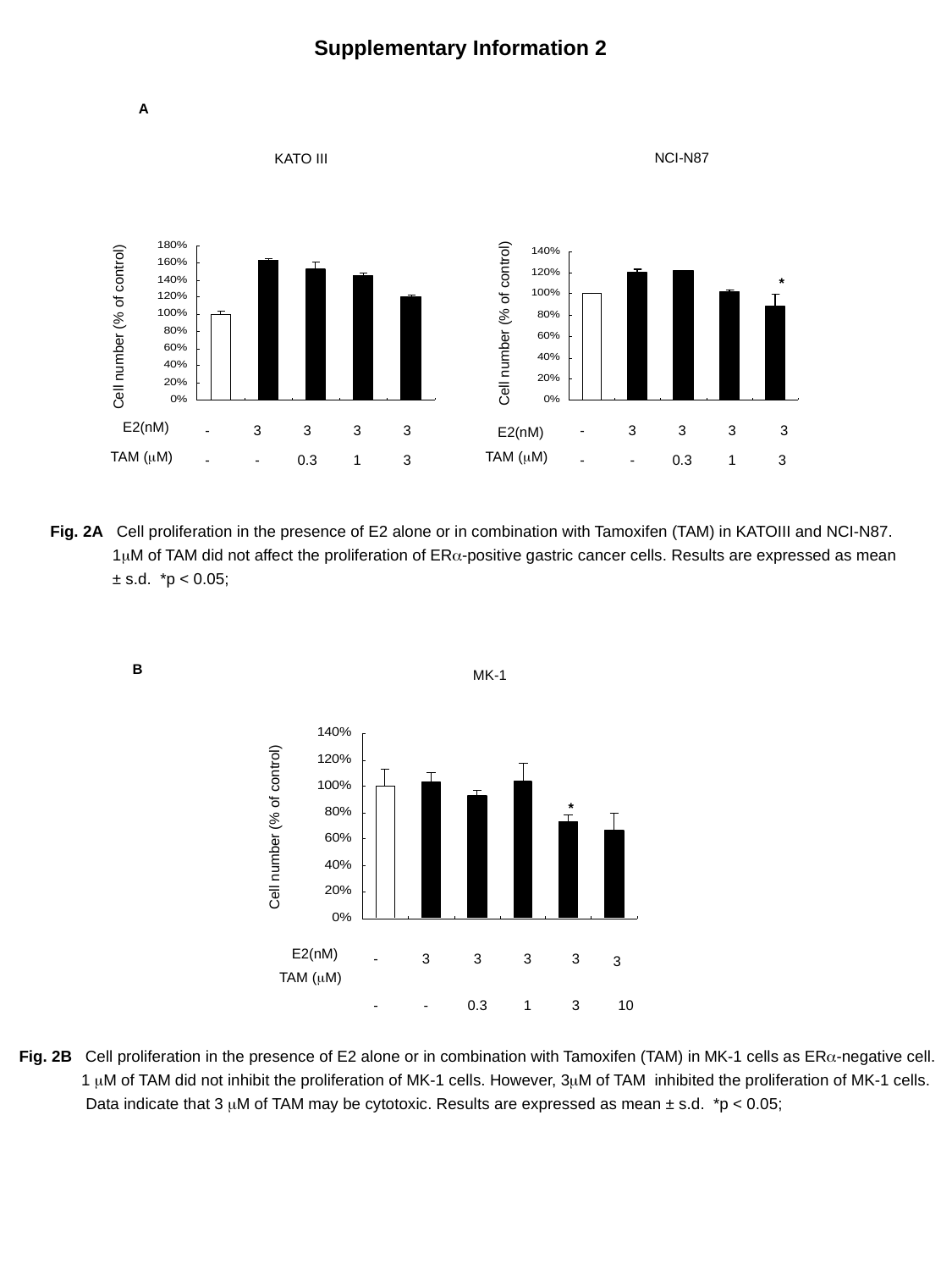

Supplementary Information 2
A
NCI-N87
KATO III
*
Cell number (% of control)
Cell number (% of control)
E2(nM)
| - | 3 | 3 | 3 | 3 |
| --- | --- | --- | --- | --- |
| - | - | 0.3 | 1 | 3 |
E2(nM)
| - | 3 | 3 | 3 | 3 |
| --- | --- | --- | --- | --- |
| - | - | 0.3 | 1 | 3 |
TAM (μM)
TAM (μM)
Fig. 2A Cell proliferation in the presence of E2 alone or in combination with Tamoxifen (TAM) in KATOIII and NCI-N87.
 1μM of TAM did not affect the proliferation of ERα-positive gastric cancer cells. Results are expressed as mean
 ± s.d. *p < 0.05;
B
MK-1
*
Cell number (% of control)
E2(nM)
| - | 3 | 3 | 3 | 3 | 3 |
| --- | --- | --- | --- | --- | --- |
| - | - | 0.3 | 1 | 3 | 10 |
TAM (μM)
Fig. 2B Cell proliferation in the presence of E2 alone or in combination with Tamoxifen (TAM) in MK-1 cells as ERα-negative cell.
 1 μM of TAM did not inhibit the proliferation of MK-1 cells. However, 3μM of TAM inhibited the proliferation of MK-1 cells.
 Data indicate that 3 μM of TAM may be cytotoxic. Results are expressed as mean ± s.d. *p < 0.05;
